# Microbial-Derived Daidzin (Eco-3) Inhibits Adipogenesis and Lipid Accumulation in Cellular and Zebrafish Models

**DOI:** 10.3390/ijms27125394

**Published:** 2026-06-15

**Authors:** Nivethasri Lakshmana Perumal, Muneer Hussain, Kyung-Bon Koo, Kil-Hwan Han, Byeong-Churl Jang

**Affiliations:** 1 Department of Molecular Medicine, College of Medicine, Keimyung University, 1095 Dalgubeoldaero, Dalseo-gu, Daegu 42601, Republic of Korea; nivi1998@naver.com (N.L.P.); muneerhussain39@naver.com (M.H.); 2 Ecowin Co., Ltd., 18, Techno jungang-daero 2-gil, Yuga-eup, Dalseong-gun, Daegu 42415, Republic of Korea; bon612@hanmail.net (K.-B.K.); han660909@naver.com (K.-H.H.)

**Keywords:** microbial-derived daidzin, lipid accumulation, AMPK, 3T3-L1, hASCs, zebrafish

## Abstract

Daidzin is a soy-derived isoflavone with reported anti-obesity effects; however, the biological activity of microbial-derived daidzin remains poorly understood. In this study, we investigated the anti-adipogenic and anti-obesity potential of microbial-derived daidzin (hereafter referred to as Eco-3) in both in vitro and in vivo models. Eco-3 significantly suppressed adipocyte differentiation and lipid accumulation in 3T3-L1 preadipocytes and human adipose-derived stem cells (hASCs) without inducing cytotoxicity. Mechanistically, Eco-3 reduced the expression of key adipogenic regulators, including PPAR-γ and C/EBP-α, and modulated lipid metabolism-related proteins such as FAS and perilipin A. In addition, Eco-3 activated AMPK signaling while inhibiting the STAT-3 and STAT-5 pathway. In zebrafish models, Eco-3 significantly reduced lipid accumulation under both normal and diet-induced obesity conditions, as demonstrated by LipidGreen2 and Oil Red O staining. Collectively, these findings suggest that Eco-3 exerts anti-obesity effects through coordinated regulation of adipogenesis and lipid metabolism.

## 1. Introduction

Obesity is a complex, multifactorial metabolic disorder characterized not only by excessive fat accumulation but also by dysregulated energy homeostasis and chronic low-grade inflammation [1,2,3]. It represents a major global health burden and is closely associated with the development of metabolic diseases, including hyperlipidemia, type 2 diabetes, cardiovascular disease, and certain cancers [1,2]. At the cellular level, sustained energy surplus disrupts metabolic balance and promotes adipocyte hypertrophy and hyperplasia, leading to excessive triglyceride (TG) storage and progressive adipose tissue dysfunction [3,4].

Adipocyte differentiation (adipogenesis) is a dynamic and multi-layered biological process that integrates transcriptional, signaling, and metabolic networks to drive the transition of fibroblast-like preadipocytes into mature, lipid-laden adipocytes. This process is orchestrated by a hierarchical cascade of transcription factors, prominently including CCAAT/enhancer-binding proteins (C/EBPs) and peroxisome proliferator-activated receptor-γ (PPAR-γ), which act as central regulators of adipocyte lineage commitment and differentiation [5]. Beyond transcriptional control, adipogenesis is fine-tuned by interconnected signaling pathways, such as the Janus kinase (JAK)/signal transducer and activator of transcription (STAT) axis, which modulate both differentiation and metabolic function [6]. Concurrently, lipid biosynthesis and storage are mediated by key lipogenic enzymes, including fatty acid synthase (FAS) and acetyl-CoA carboxylase (ACC), along with lipid droplet-associated proteins such as perilipin A, thereby facilitating triglyceride accumulation and adipocyte maturation [7,8,9]. Importantly, these processes are further coordinated by metabolic sensors and signaling hubs, including AMP-activated protein kinase (AMPK), protein kinase A (PKA), extracellular signal-regulated kinase (ERK), and cyclic AMP (cAMP), which collectively integrate energy status with adipogenic progression [10,11,12,13,14].

Zebrafish have become a widely accepted vertebrate model for metabolic research because this model offers a rapid and cost-effective platform for investigating obesity-related phenotypes and evaluating potential anti-obesity therapeutics [15]. In addition, zebrafish possess adipose tissues and molecular regulators of adipogenesis that are comparable to those of higher vertebrates, making them a useful system for studying adipose tissue development and obesity-related processes [16]. Importantly, diet-induced obesity in zebrafish recapitulates key features of mammalian obesity, including excessive lipid accumulation and metabolic dysregulation, supporting their utility for evaluating the anti-obesity effects of candidate compounds [17].

Given the complexity of adipogenesis and metabolic regulation, significant efforts have been directed toward identifying bioactive compounds capable of suppressing adipocyte differentiation and lipid accumulation. Among these, soybean-derived isoflavones, including daidzin and daidzein, have garnered considerable attention due to their anti-obesity, antioxidant, and metabolic regulatory properties [18,19,20]. However, the biological activity of daidzin has been predominantly investigated in its plant-derived form, and thus its functional potential in alternative biochemical contexts remains largely unexplored.

Recent advances in microbial biotechnology have enabled the production and structural modification of bioactive compounds through fermentation and biotransformation processes. Microbial metabolism can significantly alter the physicochemical properties and biological activities of natural compounds, thereby enhancing their functional efficacy or generating novel bioactive profiles. In particular, symbiotic bacteria such as *Xenorhabdus* (*X.*) *nematophila* are recognized for their ability to produce diverse secondary metabolites with potent biological activities. Despite these advances, the anti-obesity potential of microbially derived daidzin has not been systematically investigated.

In this study, we investigated the anti-adipogenic and anti-obesity effects of *X. nematophila*-derived daidzin (hereafter referred to as Eco-3) using both in vitro and in vivo models. Specifically, we evaluated the effects of Eco-3 on adipocyte differentiation and lipid accumulation in 3T3-L1 preadipocytes and human adipose-derived stem cells (hASCs), and further validated its efficacy in zebrafish models under both basal and diet-induced obesity conditions. In addition, we elucidated the molecular mechanisms underlying the effects of Eco-3 by analyzing key adipogenic transcription factors, lipogenic proteins, and metabolic signaling pathways.

## 2. Results

### 2.1. Eco-3 Suppresses Adipocyte Differentiation Without Cytotoxicity in 3T3-L1 Cells

**Figure 1:**
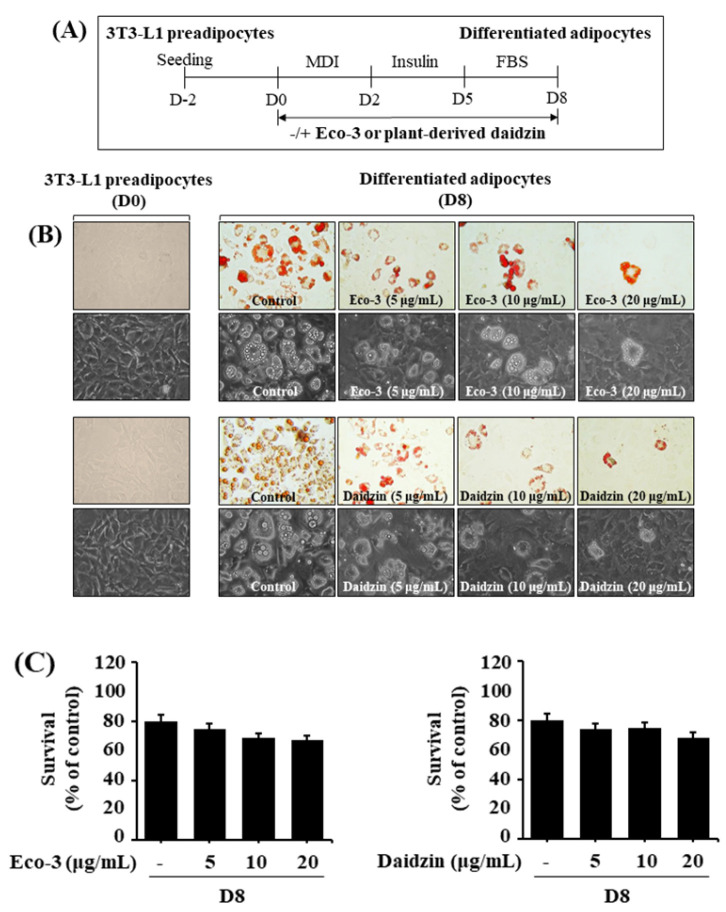
Effects of Eco-3 and plant-derived daidzin on adipocyte differentiation and lipid accumulation in 3T3-L1 cells. (**A**) Schematic illustration of adipocyte differentiation in 3T3-L1 cells. Preadipocytes were induced to differentiate using an MDI cocktail at day 0 (D0), followed by insulin-containing medium, and treated with Eco-3 (0, 5, 10, and 20 μg/mL) from D0 to D8. (**B**) Representative Oil Red O-stained and phase-contrast images of 3T3-L1 cells at D0 and D8 following treatment with Eco-3 or plant-derived daidzin. Both Eco-3 and plant-derived daidzin reduced intracellular lipid accumulation in a dose-dependent manner compared with their respective control groups. Phase-contrast images demonstrate morphological changes associated with adipocyte differentiation and lipid droplet formation. (**C**) Cell viability of 3T3-L1 cells after 8 days of treatment with Eco-3 or plant-derived daidzin. Cell viability was expressed as a percentage relative to the control group. Neither Eco-3 nor plant-derived daidzin induced significant cytotoxicity at the tested concentrations, indicating that the inhibitory effects on adipocyte differentiation were not attributable to reduced cell viability. Data are presented as mean ± SE from at least three independent experiments.

To investigate the anti-adipogenic effects of Eco-3, 3T3-L1 preadipocytes were induced to differentiate into mature adipocytes in the presence of Eco-3 (0, 5, 10, and 20 μg/mL) according to a standard differentiation protocol (Figure 1A). Untreated control cells exhibited typical adipogenic differentiation, characterized by prominent intracellular lipid droplet accumulation at day 8 (D8), as demonstrated by Oil Red O staining and phase-contrast microscopy (Figure 1B). In contrast, Eco-3 treatment markedly reduced lipid droplet formation in a dose-dependent manner. Notably, treatment with Eco-3 at 10 and 20 μg/mL resulted in a pronounced reduction in lipid accumulation compared with the control group, indicating effective suppression of adipocyte differentiation. To compare the anti-adipogenic efficacy of Eco-3 with plant-derived daidzin (commercial standard daidzin), 3T3-L1 cells were treated with equivalent concentrations of plant-derived daidzin under identical experimental conditions. Although plant-derived daidzin also reduced lipid accumulation, Eco-3 exhibited a stronger inhibitory effect on 3T3-L1 preadipocyte differentiation, particularly at higher concentrations, as evidenced by reduced Oil Red O-positive lipid droplets and altered adipocyte morphology. These findings suggest a possible role for microbial biotransformation in modulating the anti-adipogenic activity of daidzin. To determine whether the observed anti-adipogenic effects were associated with cytotoxicity, cell viability was next evaluated at D8. Both Eco-3 and plant-derived daidzin did not induce significant cytotoxicity in 3T3-L1 cells at the tested concentrations, as evidenced by the maintenance of normal cell morphology and viability (Figure 1C). These findings suggest that the inhibitory effects of Eco-3 and daidzin on adipocyte differentiation are unlikely to result from nonspecific cytotoxic effects. These results indicate that Eco-3 suppresses adipocyte differentiation and lipid accumulation in 3T3-L1 cells without inducing significant cytotoxicity. To further characterize Eco-3, HPLC analysis was performed. Eco-3 exhibited a major chromatographic peak at approximately 29 min under the applied analytical conditions, supporting the successful production and characterization of microbial-derived daidzin (Supplementary Figure S1).

### 2.2. Eco-3 Regulates Adipogenic Transcription Factors, Lipogenic Proteins, and Metabolic Signaling Pathways in Differentiated 3T3-L1 Cells

**Figure 2:**
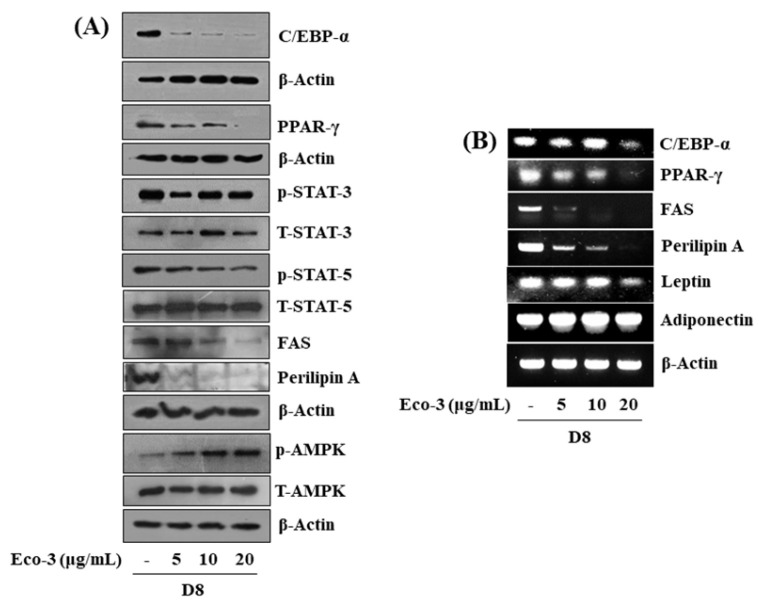
Effects of Eco-3 on adipogenic transcription factors, lipogenic proteins, and metabolic signaling pathways in differentiated 3T3-L1 cells. (**A**,**B**) 3T3-L1 cells were induced to differentiate in the presence of Eco-3 (0, 5, 10, and 20 μg/mL) from day 0 (D0) to day 8 (D8). Protein expression levels of adipogenic transcription factors, lipogenic proteins, and signaling molecules were analyzed by Western blotting (**A**), while mRNA expression levels of adipogenesis- and lipid metabolism-related genes were analyzed by RT-PCR assay (**B**). Eco-3 treatment reduced the expression of adipogenic and lipogenic markers, including PPAR-γ, C/EBP-α, FAS, and perilipin A, suppressed STAT-3 and STAT-5 phosphorylation, and increased AMPK phosphorylation in a dose-dependent manner.

To elucidate the molecular mechanisms underlying the anti-adipogenic effects of Eco-3, the expression of key adipogenic transcription factors and lipid metabolism-related proteins was analyzed in differentiated 3T3-L1 cells (D8). Western blot analysis revealed that Eco-3 treatment markedly reduced the expression of the master adipogenic regulators PPAR-γ and C/EBP-α in a dose-dependent manner (Figure 2A). Moreover, the phosphorylation levels of STAT-3 and STAT-5, which are known to promote adipogenesis, were also decreased in Eco-3-treated cells. In addition, the expression of lipogenic proteins, including FAS and perilipin A, was significantly reduced following Eco-3 treatment, indicating suppression of lipid synthesis and lipid droplet formation. Furthermore, Eco-3 increased AMPK phosphorylation, suggesting activation of energy metabolism. RT-PCR analysis further demonstrated that Eco-3 decreased the mRNA expression of PPAR-γ, C/EBP-α, FAS, perilipin A, and the adipokine leptin (Figure 2B). In contrast, Eco-3 had no significant effect on adiponectin mRNA expression. Collectively, these findings suggest that Eco-3 inhibits adipocyte differentiation and lipid accumulation by modulating the PPAR-γ-C/EBP-α axis, suppressing STAT-3/5 signaling, and activating AMPK-dependent metabolic pathway. To further investigate the temporal effects of Eco-3 during adipogenesis, early and intermediate stages of adipocyte differentiation (D2 and D5) were additionally analyzed. Eco-3 consistently reduced the expression of adipogenic and lipogenic markers while modulating STAT- and AMPK-associated signaling pathways throughout the progression of adipocyte differentiation (Supplementary Figures S2 and S3).

### 2.3. Eco-3 Inhibits Adipocyte Differentiation and Lipid Accumulation in Human Adipose-Derived Stem Cells (hASCs) Without Cytotoxicity

**Figure 3:**
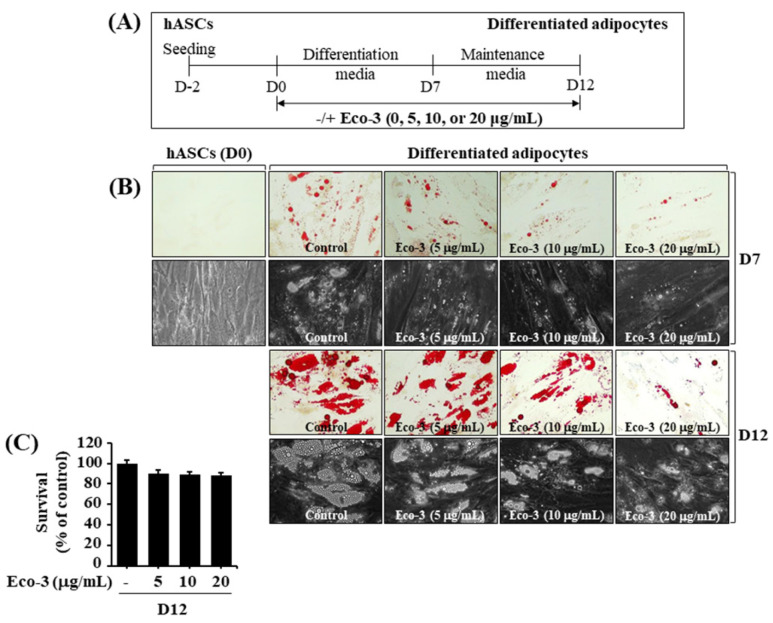
Effects of Eco-3 on adipocyte differentiation and lipid accumulation in human adipose-derived stem cells (hASCs) without cytotoxicity. (**A**) Schematic illustration of adipocyte differentiation in hASCs. (**B**) Representative Oil Red O-stained and phase-contrast images of hASCs at the preadipocyte stage (D0) and differentiated adipocyte stages (D7 and D12) following Eco-3 treatment. Control cells exhibited progressive intracellular lipid droplet accumulation during adipocyte differentiation, whereas Eco-3 treatment reduced lipid accumulation in a dose-dependent manner. Phase-contrast images demonstrate morphological changes associated with adipocyte differentiation and lipid droplet formation. (**C**) Cell viability of hASCs after 12 days of Eco-3 treatment. Cell viability was expressed as a percentage relative to the control group. Eco-3 did not induce significant cytotoxicity at the tested concentrations. Data are presented as mean ± SE from at least three independent experiments.

To validate the anti-adipogenic effects of Eco-3 in a human cell model, hASCs were induced to differentiate into adipocytes in the presence of Eco-3 (0, 5, 10, and 20 μg/mL) according to the experimental schedule shown in Figure 3A. Microscopic analysis revealed that untreated control hASCs underwent typical adipogenic differentiation, characterized by progressive intracellular lipid droplet accumulation at both day 7 (D7) and day 12 (D12), as demonstrated by Oil Red O staining and phase-contrast microscopy (Figure 3B). In contrast, Eco-3 treatment markedly reduced lipid accumulation in a dose-dependent manner at both differentiation stages. Notably, the inhibitory effect of Eco-3 was more pronounced at D12, indicating sustained suppression of adipocyte maturation and lipid deposition during late-stage adipogenesis. To determine whether the observed anti-adipogenic effects were associated with cytotoxicity, cell viability was assessed at D12. Eco-3 treatment did not significantly affect cell survival at any tested concentration, with cell viability remaining above approximately 85–95% of control levels (Figure 3C). These findings indicate that Eco-3 suppresses adipocyte differentiation and lipid accumulation in hASCs without inducing significant cytotoxicity. Collectively, these results demonstrate that Eco-3 effectively inhibits adipogenesis in hASCs, consistent with its anti-adipogenic effects observed in 3T3-L1 cells, thereby supporting the potential translational relevance of Eco-3 in human adipose-associated metabolic conditions.

### 2.4. Eco-3 Suppresses Adipogenic and Lipogenic Signaling Pathways During Adipocyte Differentiation in hASCs

**Figure 4:**
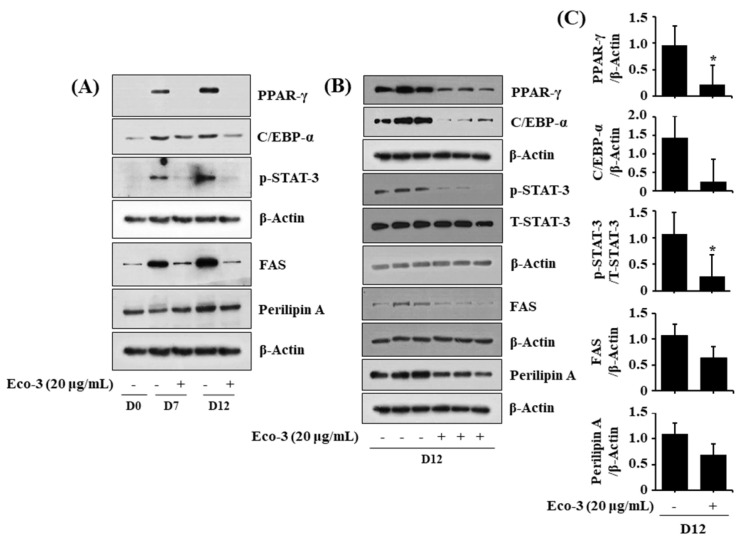
Effects of Eco-3 on adipogenic transcription factors, lipogenic proteins, and STAT3 signaling during adipocyte differentiation in hASCs. (**A**) hASCs were induced to differentiate in the presence or absence of Eco-3 (20 μg/mL), and protein expression levels were analyzed at different stages of adipocyte differentiation (D0, D7, and D12). Whole-cell lysates collected at each time point were subjected to immunoblot analysis. (**B**) Representative immunoblot images from triplicate experiments performed at D12. (**C**) Densitometric analysis of the immunoblotting data shown in (**B**). Eco-3 treatment significantly reduced the expression of adipogenic transcription factors and lipogenic proteins, as well as STAT-3 phosphorylation, compared with untreated control cells. Data are presented as mean ± SE. * *p* < 0.05 versus the Eco-3–untreated control at the corresponding time point.

To further investigate the molecular mechanisms underlying the anti-adipogenic effects of Eco-3 in human cells, the expression of key adipogenic transcription factors, lipogenic proteins, and signaling molecules was analyzed in hASCs at different stages of adipocyte differentiation (D0, D7, and D12). In untreated control cells, the expression and phosphorylation of PPAR-γ, C/EBP-α, and STAT-3, as well as FAS and perilipin A, progressively increased during adipocyte differentiation from D0 to D12 (Figure 4A), reflecting the normal progression of adipocyte commitment and maturation. At the intermediate stage of differentiation (D7), Eco-3 treatment markedly reduced the expression of PPAR-γ, C/EBP-α, and FAS, suggesting suppression of adipogenic commitment and lipid synthesis. In contrast, perilipin A expression was not markedly affected at this stage. At the late stage of differentiation (D12), Eco-3 further suppressed the expression and phosphorylation of PPAR-γ, C/EBP-α, STAT-3, FAS, and perilipin A, indicating sustained inhibition of adipocyte maturation and lipid accumulation. Furthermore, triplicate experiments performed at D12 consistently demonstrated that Eco-3 significantly reduced the expression of these adipogenic transcription factors and lipogenic proteins compared with untreated control cells (Figure 4B,C). Collectively, these findings indicate that Eco-3 suppresses adipocyte differentiation and maturation in hASCs through coordinated inhibition of adipogenic and lipogenic signaling pathways.

### 2.5. Eco-3 Reduces Lipid Accumulation in Zebrafish Larvae

**Figure 5:**
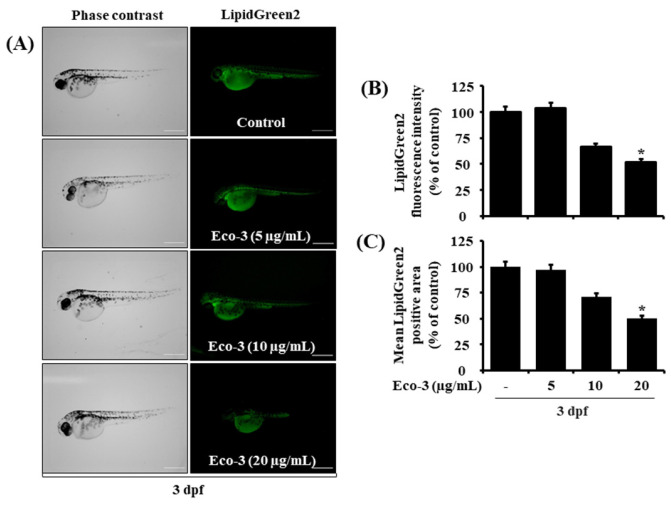
Effects of Eco-3 on lipid accumulation in zebrafish larvae. (**A**) Zebrafish larvae were treated with Eco-3 (0, 5, 10, and 20 μg/mL), and lipid accumulation was evaluated at 3 days post-fertilization (3 dpf) using LipidGreen2 staining. Representative phase-contrast and fluorescence images are shown, Scale bar = 100 μm. Control larvae exhibited strong LipidGreen2 fluorescence, indicating substantial lipid accumulation, whereas Eco-3-treated larvae displayed a dose-dependent reduction in fluorescence intensity. (**B**) Quantification of LipidGreen2 fluorescence intensity, expressed as a percentage relative to the control group. (**C**) Quantification of the mean LipidGreen2-positive area, expressed as a percentage relative to the control group. Eco-3 treatment significantly reduced both fluorescence intensity and lipid-positive area compared with untreated control larvae. Data are presented as mean ± SE from at least three independent experiments. * *p* < 0.05 versus control.

To evaluate the anti-obesity effects of Eco-3 in vivo, lipid accumulation was assessed in zebrafish larvae at 3 days post-fertilization (3 dpf) using LipidGreen2 staining. Representative fluorescence images revealed strong LipidGreen2 signals in control larvae, indicating substantial lipid accumulation, particularly in the yolk sac region (Figure 5A). In contrast, Eco-3-treated larvae exhibited a dose-dependent reduction in LipidGreen2 fluorescence intensity, with more pronounced decreases observed at 10 and 20 μg/mL. Quantitative analysis further supported these observations. Eco-3 treatment significantly reduced LipidGreen2 fluorescence intensity compared with untreated control larvae (Figure 5B). In addition, the LipidGreen2-positive area was markedly decreased in Eco-3-treated groups, indicating reduced overall lipid accumulation in zebrafish larvae (Figure 5C). Notably, Eco-3 treatment did not induce obvious morphological abnormalities or developmental defects at the tested concentrations. Collectively, these findings demonstrate that Eco-3 effectively suppresses lipid accumulation in zebrafish larvae in a dose-dependent manner, supporting its anti-obesity potential in vivo.

### 2.6. Eco-3 Attenuates Diet-Induced Lipid Accumulation in a Zebrafish Obesity Model

**Figure 6:**
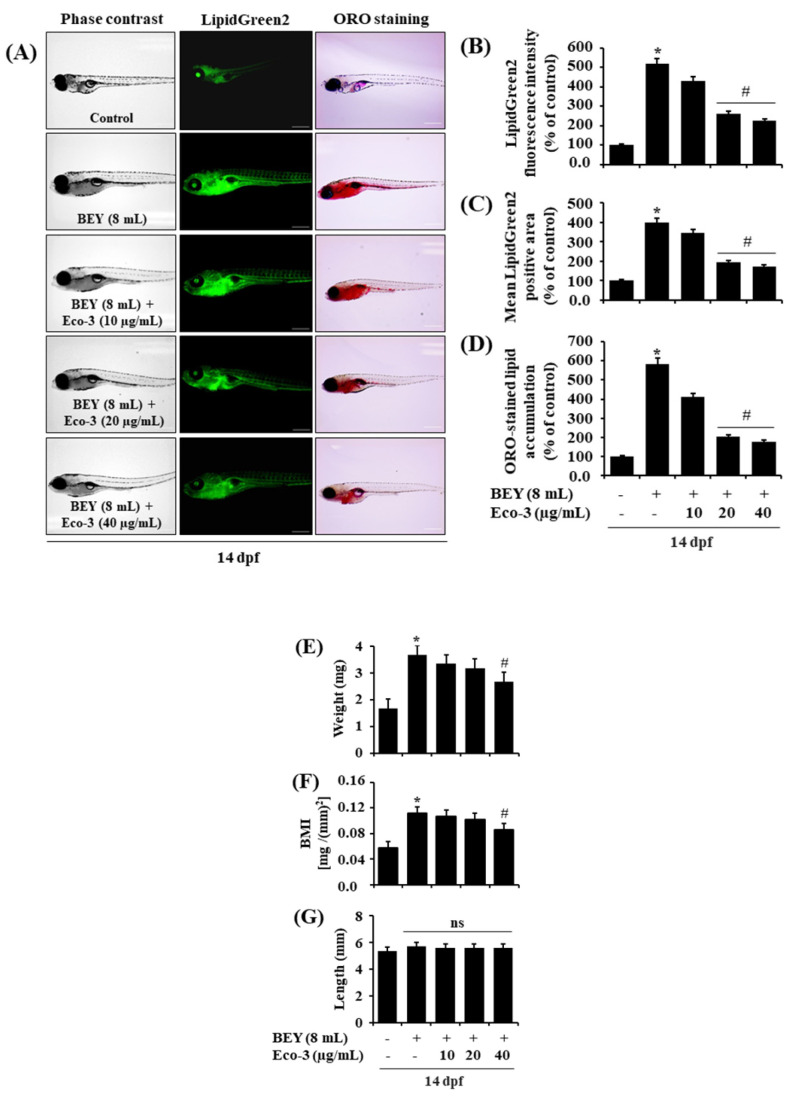
Effects of Eco-3 on diet-induced lipid accumulation and body composition in a zebrafish obesity model. (**A**–**G**) A diet-induced obesity model was established in zebrafish larvae by boiled egg yolk (BEY) feeding, followed by treatment with Eco-3 (10, 20, and 40 μg/mL). Analyses were performed at 14 dpf. Representative phase-contrast, LipidGreen2 fluorescence, and Oil Red O-stained images of zebrafish larvae are shown, Scale bar = 100 μm (**A**). Quantitative analysis of LipidGreen2 fluorescence intensity and lipid-positive area, expressed as percentages relative to the control group (**B,C**). Quantification of Oil Red O-positive staining in zebrafish larvae (**D**). Quantitative analysis of body weight (**E**), BMI (**F**), and body length (**G**). Eco-3 treatment dose-dependently reduced diet-induced lipid accumulation, body weight, and BMI, while exerting minimal effects on body length. Data are presented as mean ± SE from at least three independent experiments. ns, not significant; * *p* < 0.05 versus the BEY-treated group. # *p* < 0.05 versus the obesity (BEY-fed) positive control group.

To further evaluate the anti-obesity effects of Eco-3 under pathological conditions, a diet-induced obesity model was established in zebrafish using boiled egg yolk (BEY) feeding. Lipid accumulation was assessed at 14 dpf using LipidGreen2 and Oil Red O staining. Representative images revealed that BEY-fed zebrafish exhibited markedly increased lipid accumulation compared with untreated control larvae, as evidenced by enhanced LipidGreen2 fluorescence and Oil Red O-positive staining (Figure 6A). In contrast, Eco-3 treatment dose-dependently reduced lipid accumulation in BEY-fed zebrafish. Notably, treatment with Eco-3 at 20 and 40 μg/mL markedly attenuated both fluorescence intensity and lipid-positive staining compared with the BEY-only group (Figure 6B–D), indicating effective suppression of diet-induced lipid accumulation. These findings demonstrate that Eco-3 not only inhibits basal lipid accumulation but also effectively attenuates pathological lipid deposition induced by excessive dietary lipid intake. Furthermore, the dose-dependent reduction in lipid accumulation supports the anti-obesity potential of Eco-3 under metabolically challenged conditions. In addition, compared with untreated control larvae, BEY-fed zebrafish exhibited increased body weight (Figure 6E) and body mass index (BMI) (Figure 6F), confirming successful induction of an obese phenotype. In contrast, body length showed relatively minor changes among the experimental groups (Figure 6G), suggesting that the BEY-induced increase in BMI was primarily attributable to increased body weight rather than generalized body growth. Importantly, Eco-3 treatment significantly attenuated the BEY-induced increases in body weight and BMI in a dose-dependent manner while exerting minimal effects on body length. These findings indicate that Eco-3 specifically reduces adiposity and lipid accumulation without substantially affecting normal developmental growth in zebrafish larvae.

## 3. Discussion

In the present study, we investigated the anti-adipogenic and anti-obesity effects of microbial-derived daidzin (Eco-3) using integrated in vitro and in vivo experimental models. Eco-3 consistently suppressed adipocyte differentiation and lipid accumulation in 3T3-L1 preadipocytes and human adipose-derived stem cells (hASCs), while also attenuating lipid deposition in zebrafish under both basal and diet-induced obesity conditions. Mechanistically, Eco-3 downregulated key adipogenic regulators, including PPAR-γ and C/EBP-α, reduced the expression of lipogenic proteins such as FAS and perilipin A, suppressed STAT3/STAT5 signaling, and enhanced AMPK-associated metabolic signaling. To the best of our knowledge, this study is the first to demonstrate the anti-adipogenic and anti-obesity effects of microbial-derived daidzin using integrated cellular and zebrafish obesity models. Collectively, these findings indicate that Eco-3 functions as a potent regulator of adipogenesis and lipid metabolism across multiple biological systems.

Adipogenesis is a highly coordinated biological process involving sequential activation of transcriptional programs and metabolic signaling pathways that ultimately drive the conversion of fibroblast-like preadipocytes into mature lipid-laden adipocytes [21,22]. Among the transcriptional regulators involved in this process, PPAR-γ and C/EBP-α are recognized as master regulators of adipocyte differentiation and adipocyte-specific gene expression [23,24]. Activation of these transcription factors promotes lipid uptake, triglyceride synthesis, and adipocyte maturation, thereby contributing to adipose tissue expansion during obesity. In the present study, Eco-3 markedly suppressed the expression of PPAR-γ and C/EBP-α in differentiated adipocytes, indicating that Eco-3 interferes with the core transcriptional machinery governing adipogenic differentiation. These findings are consistent with previous reports demonstrating that inhibition of PPAR-γ and C/EBP-α effectively attenuates adipocyte differentiation and obesity-associated metabolic dysfunction [25].

In addition to adipogenic transcription factors, lipogenesis and lipid droplet formation are essential processes during adipocyte maturation. FAS is a key lipogenic enzyme responsible for de novo fatty acid synthesis, whereas perilipin A plays a critical role in lipid droplet stabilization and triglyceride storage [26,27]. Increased expression of these proteins is closely associated with enhanced lipid accumulation and adipocyte hypertrophy. In the present study, Eco-3 significantly reduced the expression of FAS and perilipin A at both the protein and mRNA levels, suggesting suppression of lipid synthesis and lipid droplet formation. These observations indicate that Eco-3 not only inhibits adipocyte differentiation but also reduces the lipid storage capacity of adipocytes. Such coordinated suppression of adipogenic and lipogenic pathways likely contributes to the anti-obesity effects observed in both cellular and zebrafish models.

An important finding of the present study is that Eco-3 exhibited stronger anti-adipogenic activity than plant-derived daidzin. Although plant-derived daidzin and related soybean isoflavones have previously been reported to exert anti-obesity and metabolic regulatory activities [28,29], most prior studies focused primarily on naturally isolated plant-derived compounds. In contrast, relatively little is known regarding the biological activity of microbially transformed daidzin. Recent advances in microbial biotechnology have demonstrated that microbial fermentation and biotransformation can alter the physicochemical and biological properties of natural compounds, resulting in enhanced efficacy or generation of distinct bioactive metabolites [30,31]. Microbial metabolism may influence molecular stability, solubility, glycosylation status, or metabolic conversion efficiency, thereby modulating biological activity. In this context, the present findings suggest that microbial transformation using *X. nematophila* may enhance the anti-adipogenic potential of daidzin and provide a promising strategy for generating functionally improved bioactive compounds.

Another notable observation in this study is the stage-dependent modulation of adipogenic signaling by Eco-3. Supplementary analyses demonstrated that Eco-3 regulated adipogenic and metabolic signaling pathways not only at the terminal differentiation stage (D8), but also during the early and intermediate stages of adipogenesis (D2 and D5). In particular, Eco-3 suppressed adipogenic markers and STAT signaling while enhancing AMPK-associated signaling throughout adipocyte differentiation (Supplementary Figures S2 and S3). These findings suggest that Eco-3 acts throughout the adipogenic process rather than exclusively affecting terminal lipid accumulation. Such temporal regulation may represent an important feature underlying the sustained anti-adipogenic effects of Eco-3.

STAT signaling pathways are increasingly recognized as important regulators of adipogenesis and metabolic homeostasis. Previous studies have demonstrated that STAT-3 and STAT-5 activation promotes adipocyte differentiation and lipid accumulation through regulation of adipogenic gene expression [32,33]. In particular, STAT-3 signaling has been implicated in adipocyte maturation, inflammation-associated metabolic dysfunction, and obesity-related signaling networks [34]. In the present study, Eco-3 consistently reduced STAT-3 and STAT-5 phosphorylation without markedly affecting total protein levels, suggesting selective suppression of adipogenic STAT activation. Similar regulatory patterns were observed during early adipocyte differentiation stages, indicating that Eco-3 may interfere with adipogenic signaling cascades from the initiation phase of differentiation.

Previous studies have also reported that daidzin modulates STAT-3-associated signaling pathways in various pathological conditions, including cancer models [35,36]. These findings, together with the present results, suggest that regulation of STAT signaling may represent a broader biological activity associated with daidzin-derived compounds. Because JAK and Src family kinases are important upstream regulators of STAT-3 activation [37], further studies will be required to determine whether Eco-3 modulates adipogenesis through upstream JAK/Src-dependent signaling pathways.

Another important mechanistic finding in this study is the activation of AMPK signaling by Eco-3. AMPK is a central metabolic sensor that regulates cellular energy homeostasis, lipid metabolism, mitochondrial activity, and glucose utilization [38]. Activation of AMPK suppresses adipogenesis and lipogenesis while promoting fatty acid oxidation [39]. Numerous anti-obesity compounds exert metabolic regulatory effects through activation of AMPK-associated pathways [40]. In this study, Eco-3 increased AMPK phosphorylation in differentiated adipocytes and also enhanced ACC phosphorylation during adipogenesis. ACC is a downstream target of AMPK and a key enzyme involved in fatty acid synthesis. Phosphorylation-mediated inhibition of ACC suppresses lipogenesis and promotes metabolic remodeling [41]. Therefore, the observed increase in p-ACC further supports the notion that Eco-3 activates AMPK-dependent metabolic signaling pathways. Taken together, these findings indicate that Eco-3 coordinately regulates adipogenic transcription factors and metabolic signaling pathways to suppress adipocyte differentiation and lipid accumulation.

The zebrafish experiments further strengthen the physiological and translational relevance of the present findings. Zebrafish have emerged as a valuable vertebrate model for obesity and metabolic disease research due to their conserved lipid metabolism pathways, optical transparency, and suitability for rapid in vivo lipid analysis [42,43]. In the present study, Eco-3 significantly reduced LipidGreen2 fluorescence intensity and Oil Red O-positive lipid accumulation in zebrafish larvae. Furthermore, in the boiled egg yolk (BEY)-induced obesity model, Eco-3 attenuated increases in body weight and body mass index (BMI). Importantly, Eco-3 exerted minimal effects on body length, suggesting that its anti-obesity effects were not attributable to nonspecific developmental toxicity or generalized growth suppression. This distinction is particularly important because reductions in body size can sometimes falsely appear as anti-obesity effects in zebrafish models. Therefore, preservation of normal body length strongly supports the specificity of Eco-3-mediated anti-adipogenic and lipid-lowering activity. The consistent anti-adipogenic effects observed across murine, human, and zebrafish models further support the translational potential of Eco-3 as a candidate metabolic regulatory compound.

The inclusion of hASC experiments also strengthens the translational significance of this study. Although 3T3-L1 cells are widely used as a murine adipogenesis model, hASCs provide a more clinically relevant human adipogenic system [44]. Eco-3 consistently inhibited adipocyte differentiation and lipid accumulation in both murine and human cellular models, indicating that its anti-adipogenic activity is not restricted to a single species or cellular context. This cross-model reproducibility further supports the therapeutic potential of Eco-3 as a metabolically active bioactive compound.

Despite these promising findings, several limitations of the present study should be acknowledged. First, although Eco-3 exhibited relatively enhanced anti-adipogenic activity compared with plant-derived daidzin, the precise biochemical alterations induced by microbial biotransformation were not fully characterized. Additional analytical studies will therefore be required to further define the active components and molecular properties of Eco-3. Second, although Eco-3 modulated STAT- and AMPK-associated signaling pathways, direct mechanistic validation using pathway-specific inhibitors or genetic approaches was not performed. Further studies will be necessary to elucidate the genetic, molecular, and enzymatic pathways underlying microbial transformation of Eco-3-mediated anti-adipogenic effects and clarify how these biochemical and structural modifications influence its biological activity. Third, while zebrafish models provide a rapid and useful in vivo platform for obesity research, additional validation in mammalian obesity models will be important to further evaluate the therapeutic potential and safety of Eco-3.

In conclusion, the present study demonstrates that microbial-derived daidzin (Eco-3) suppresses adipogenesis and lipid accumulation through coordinated regulation of adipogenic transcription factors, lipogenic proteins, and metabolic signaling pathways. Eco-3 consistently exhibited anti-obesity effects in murine, human, and zebrafish models, supporting its translational potential as a metabolically active bioactive compound. These findings further highlight microbial biotransformation as a promising strategy for enhancing the functional efficacy of natural products.

## 4. Materials and Methods

### 4.1. Chemicals and Reagents

Stem cell differentiation media (DM-2 and AM-1) were obtained from Zenbio (Research Triangle Park, NC, USA). Enhanced chemiluminescence (ECL) reagent was purchased from Advansta (Menlo Park, CA, USA), and the Pierce BCA Protein Assay Kit was purchased from Thermo Scientific (Rockford, IL, USA). 3-isobutyl-1-methylxanthine (IBMX), dexamethasone, insulin, and plant-derived daidzin (commercial standard daidzin) were obtained from Sigma (St. Louis, MO, USA). A comprehensive list of the antibodies used in this study is listed in Table S1.

### 4.2. Production, Isolation, and Identification of Eco-3 from Xenorhabdus nematophila

*Xenorhabdus (X.) nematophila*, a symbiotic bacterium known to produce diverse bioactive metabolites, was cultured in liquid medium at 28 °C under shaking conditions for microbial biotransformation and metabolite production. After incubation, the culture broth was centrifuged, and the supernatant was extracted with an organic solvent. The solvent extract was concentrated under reduced pressure and fractionated by silica gel and reversed-phase C18 chromatography. The target fraction was further purified by semi-preparative HPLC.

### 4.3. Cell Culture and Adipogenic Differentiation

Murine 3T3-L1 preadipocytes were obtained from ATCC (Manassas, VA, USA) and maintained in Dulbecco’s Modified Eagle Medium (DMEM; Gibco, Gaithersburg, MD, USA) supplemented with 1% penicillin-streptomycin (Welgene, Daegu, Republic of Korea) and 10% heat-inactivated fetal calf serum (FCS) (Gibco, Grand Island, NY, USA). To induce adipogenic differentiation, confluent 3T3-L1 preadipocytes were cultured in DMEM containing 10% heat-inactivated fetal bovine serum (FBS; Welgene, Daegu, Republic of Korea) supplemented with a standard adipogenic induction cocktail consisting of 0.5 mM 3-isobutyl-1-methylxanthine (IBMX), 0.5 μM dexamethasone, and 5 μg/mL insulin (MDI). Eco-3 or plant-derived daidzin was administered at the indicated concentrations (5, 10, and 20 μg/mL) throughout the differentiation period, with untreated cells used as controls. After 48 h of MDI induction (D2), the medium was replaced with DMEM supplemented with 10% FBS and 5 μg/mL insulin in the presence or absence of Eco-3 or plant-derived daidzin. Thereafter, the cells were maintained in DMEM containing 10% FBS, and the medium was refreshed every 2 days until day 8 (D8). For temporal analysis of adipogenic differentiation, samples were additionally collected at D2 and D5. By D8, control cells exhibited characteristic mature adipocyte morphology with prominent intracellular lipid droplet accumulation.

Human adipose-derived stem cells (hASCs) were isolated from abdominal subcutaneous adipose tissue obtained from female patients admitted to Keimyung University Dongsan Hospital (KUDH), Daegu, Republic of Korea, as previously described [45]. The study protocol was approved by the Institutional Review Board of KUDH (No. 2021-02-063-018), and written informed consent was obtained from all patients. For adipogenic differentiation, hASCs were cultured in adipogenic differentiation medium (DM-2) from day 0 to day 6 and subsequently maintained in adipocyte maintenance medium (AM-1) from day 7 to day 14 (ZenBio, Research Triangle Park, NC, USA) in the presence or absence of Eco-3 (5, 10, and 20 μg/mL).

### 4.4. Lipid Droplet Staining

At the indicated time points during differentiation of 3T3-L1 cells and hASCs, control and Eco-3- or plant-derived daidzin-treated cells were washed twice with phosphate-buffered saline (PBS) and fixed with 4% paraformaldehyde for 20 min at room temperature (RT). The fixed cells were subsequently stained with Oil Red O solution (Abcam, Cambridge, UK) for 1 h at RT and washed twice with distilled water. Lipid accumulation and intracellular lipid droplets were visualized and photographed under a light microscope (Nikon, Tokyo, Japan).

### 4.5. Analysis of Cell Counting

3T3-L1 cells and hASCs were cultured under the differentiation conditions described above in the presence or absence of Eco-3 or plant-derived daidzin. Cell viability during adipocyte differentiation was assessed by trypan blue exclusion assay on day 8 (D8) for 3T3-L1 cells and day 12 (D12) for hASCs. Viable cells with intact plasma membranes excluded trypan blue dye, whereas non-viable cells with compromised membranes were stained and counted under a light microscope. Cell viability assays were performed in triplicate, and the results are presented as mean ± standard error (SE) from three independent experiments.

### 4.6. Isolation of Whole-Cell Lysates

At the indicated time points, 3T3-L1 cells and hASCs were washed twice with phosphate-buffered saline (PBS) and lysed using modified radioimmunoprecipitation assay (RIPA) buffer containing 50 mM Tris-HCl (pH 7.4), 150 mM NaCl, 0.1% sodium dodecyl sulfate (SDS), 0.25% sodium deoxycholate, 1% Triton X-100, 1% Nonidet P-40, 1 mM EDTA, 1 mM EGTA, and 1× protease inhibitor cocktail. The resulting cell lysates were collected and used for protein analysis. Protein concentrations were determined using a Pierce BCA Protein Assay Kit (Thermo Fisher Scientific, Waltham, MA, USA).

### 4.7. Immunoblot Analysis

Equal amounts of protein (30 μg) were separated by 10% sodium dodecyl sulfate–polyacrylamide gel electrophoresis (SDS-PAGE) and transferred onto polyvinylidene difluoride (PVDF) membranes (Millipore, Billerica, MA, USA). The membranes were washed with Tris-buffered saline containing 0.05% Tween-20 (TBST; 10 mM Tris-HCl, 150 mM NaCl, pH 7.5) and blocked with 5% non-fat dry milk in TBST for 3 h at RT. The membranes were subsequently incubated overnight at 4 °C with the primary antibodies listed in Table S1. After washing three times with TBST, the membranes were incubated with horseradish peroxidase (HRP)-conjugated secondary antibodies (anti-mouse IgG or anti-rabbit IgG) for 2 h at RT. Following three additional washes with TBST, immunoreactive bands were visualized using enhanced chemiluminescence (ECL) reagents. Equal protein loading was confirmed by β-actin expression.

### 4.8. Conventional RT-PCR

Total RNA was extracted from 3T3-L1 cells using RNAiso Plus reagent (TaKaRa, Kusatsu, Shiga, Japan) according to the manufacturer’s instructions. Three micrograms of total RNA were reverse-transcribed into complementary DNA (cDNA) using random hexamer primers and reverse transcriptase. PCR amplification was performed using gene-specific primers listed in Table S2.

### 4.9. Zebrafish Husbandry

Wild-type zebrafish (AB strain) were maintained in a continuous-flow aquatic system at 26–28.5 °C under a 14 h light/10 h dark photoperiod and fed twice daily. For embryo collection, adult female and male zebrafish were placed in spawning tanks at a 1:1 ratio and separated overnight by a divider. The following morning, the divider was removed to allow mating, and fertilized embryos were collected and transferred to Petri dishes (60 embryos per dish) containing E3 embryo medium. Embryos and larvae were maintained at 26 °C throughout the experimental period. All animal experiments were approved by the Institutional Animal Care and Use Committee (IACUC) of Keimyung University (IACUC No. KM2025-016) and conducted in accordance with the National Institutes of Health Guide for the Care and Use of Laboratory Animals.

### 4.10. Boiled Egg Yolk (BEY)-Induced Obesity Zebrafish Model

At 5 days post-fertilization (dpf), zebrafish larvae exhibiting normal development and physiological fitness were selected for experiments. At 6 dpf, larvae were randomly divided into experimental groups, with 20 larvae per condition. Larvae were fed either a standard feeding diet (StF) or a boiled egg yolk (BEY)-based obesogenic diet to induce excessive lipid accumulation and obesity. The standard feeding group received a commercially available Artemia-based diet, whereas the BEY group was fed a BEY suspension prepared by homogenizing 1 g of boiled chicken egg yolk in 15 mL of E3 medium, as previously described. Larvae in the BEY-fed group received 8 mL of the BEY suspension twice daily for 7 days (from 6 to 13 dpf), resulting in increased lipid accumulation and an obese phenotype. For Eco-3 treatment, zebrafish larvae were exposed to Eco-3 (10, 20, or 40 μg/mL) in E3 medium throughout the experimental period, and the treatment solution was refreshed daily. Control larvae were maintained in E3 medium without Eco-3 treatment.

### 4.11. LipidGreen2 Staining

LipidGreen2 staining was performed to evaluate lipid accumulation in zebrafish larvae, as previously described. Briefly, LipidGreen2 solution was prepared in E3 medium at a final concentration of 1 μM. Following staining, larvae were washed three times for 10 min each with fresh E3 medium. During the staining and washing procedures, larvae were protected from light. Larvae were anesthetized by hypothermic shock and mounted in 3% methylcellulose for imaging. Fluorescence images were captured using an inverted fluorescence microscope (Olympus Life Science, Shinjuku, Tokyo, Japan).

### 4.12. Statistical Analysis

All experiments were performed in triplicate and repeated independently at least three times. Data are presented as mean ± standard error (SE). Statistical analyses were performed using one-way analysis of variance (ANOVA) followed by Dunnett’s post hoc test using SPSS software version 11.5 (SPSS Inc., Chicago, IL, USA). Differences were considered statistically significant at *p* < 0.05.

## Data Availability

The data that support the findings of this study are available from the corresponding author upon reasonable request.

## Supplementary Materials

The following supporting information can be downloaded at: https://www.mdpi.com/article/10.3390/ijms27125394/s1.
