# Supplementary material for: Microbial-Derived Daidzin (Eco-3) Inhibits Adipogenesis and Lipid Accumulation in Cellular and Zebrafish Models
Source: Int J Mol Sci. 2026 Jun 15;27(12):5394. doi: 10.3390/ijms27125394 (PMC13299301; doi:10.3390/ijms27125394)
Supplement: Supplementary file 1 [file ijms-27-05394-s001.zip › Table 1.pdf]

Table S1: Antibodies used for Immunoblotting Analysis

Antibodies	Dilution used	Source	Catalog no.
C/EBP-α	1:2,000	Santa Cruz Biotechnology	sc-61
PPAR-γ	1:2,000	Santa Cruz Biotechnology	sc-7272
p-STAT-3	1:2,000	Santa Cruz Biotechnology	sc-8059
T-STAT-3	1:2,000	Santa Cruz Biotechnology	sc-8019
p-STAT-5	1:2,000	Santa Cruz Biotechnology	sc-81524
T-STAT-5	1:2,000	Santa Cruz Biotechnology	sc-1656
Perilipin A	1:2,000	Biovision	#3948-200
FAS	1:2,000	BD Biosciences	#610962
p-AMPK (T172)	1:2,000	Cell signaling	#2535
T-AMPK	1:2,000	Cell signaling	#2793
p-ACC (S79)	1:2,000	Cell signaling	#3661
T-ACC	1:2,000	Cell signaling	#3662
β-actin	1:10,000	Sigma	A5441
Goat anti-rabbit IgG-HRP	1:2,000	Jackson ImmunoResearch Laboratories	111-035-045
Goat anti-mouse IgG-HRP	1:2,000	Jackson ImmunoResearch Laboratories	115-035-062
