# Supplementary material for: Microbial-Derived Daidzin (Eco-3) Inhibits Adipogenesis and Lipid Accumulation in Cellular and Zebrafish Models
Source: Int J Mol Sci. 2026 Jun 15;27(12):5394. doi: 10.3390/ijms27125394 (PMC13299301; doi:10.3390/ijms27125394)
Supplement: Supplementary file 1 [file ijms-27-05394-s001.zip › Table 2.pdf]

Table S2: The list of PCR Analysis Primer Sequences and its Conditions

Primer	Sequences	Denaturation	Annealing	Extension
C/EBP-α	5′-TTACAACAGGCCAGGTTTCC-3′<br>5′-CTCTGGGATGGATCGATTGT-3′	95°C, 30 s	62°C, 30 s	72°C, 30 s
PPAR-γ	5′-GGTGAAACTCTGGGAGATTC-3′<br>5′-CAACCATTGGGTCAGCTCTC-3′	95°C, 30 s	53°C, 30 s	72°C, 30 s
FAS	5′-TTGCTGGCACTACAGAATGC-3′<br>5′-AACAGCCTCAGAGCGACAAT-3′	95°C, 15 s	55°C, 40 s	68°C, 45 s
Perilipin A	5′-TTCTCGACACACCATGGAAACC-3′<br>5′-CACGTTATCCGTAACACCCTTCA-3′	95°C, 1 min	55°C, 1 min	72°C, 1 min
Adiponectin	5′-GGAGATGCAGGTCTTCTTGGT-3′<br>5′- TCCTGATACTGGTCGTAGGTGAA-3′	95°C, 1 min	53°C, 1 min	72°C, 1 min
Leptin	5′-CCAAAACCCTCATCAAGACC-3′<br>5′-CTCAAAGCCACCACCTCTGT-3′	95°C, 1 min	57°C, 1 min	72°C, 1 min
β-actin	5′-TCATGAAGTGTGACGTTGACATCCGT-3′<br>5′-CCTAGAAGCATTTGCGGTGCACGATG-3′	95°C, 30 s	57°C, 30 s	72°C, 1 min
