# Supplementary figures and images for: Microbial-Derived Daidzin (Eco-3) Inhibits Adipogenesis and Lipid Accumulation in Cellular and Zebrafish Models
Source: Int J Mol Sci. 2026 Jun 15;27(12):5394. doi: 10.3390/ijms27125394 (PMC13299301; doi:10.3390/ijms27125394)

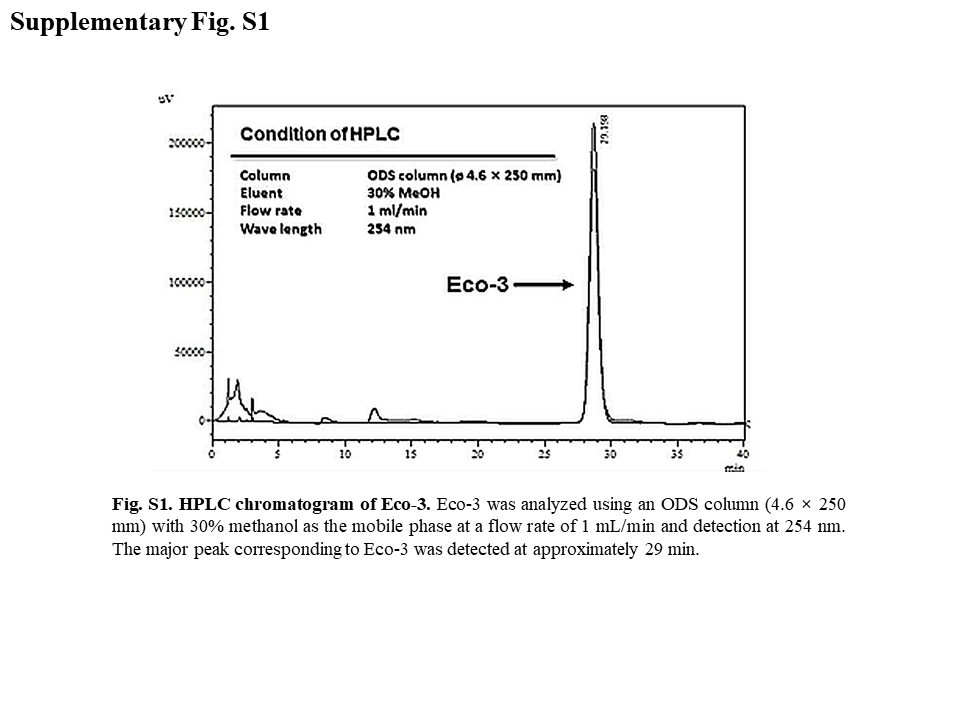

Supplement: Supplementary file 1 [file ijms-27-05394-s001.zip › Supplementary Figures/Fig. S1.TIF]

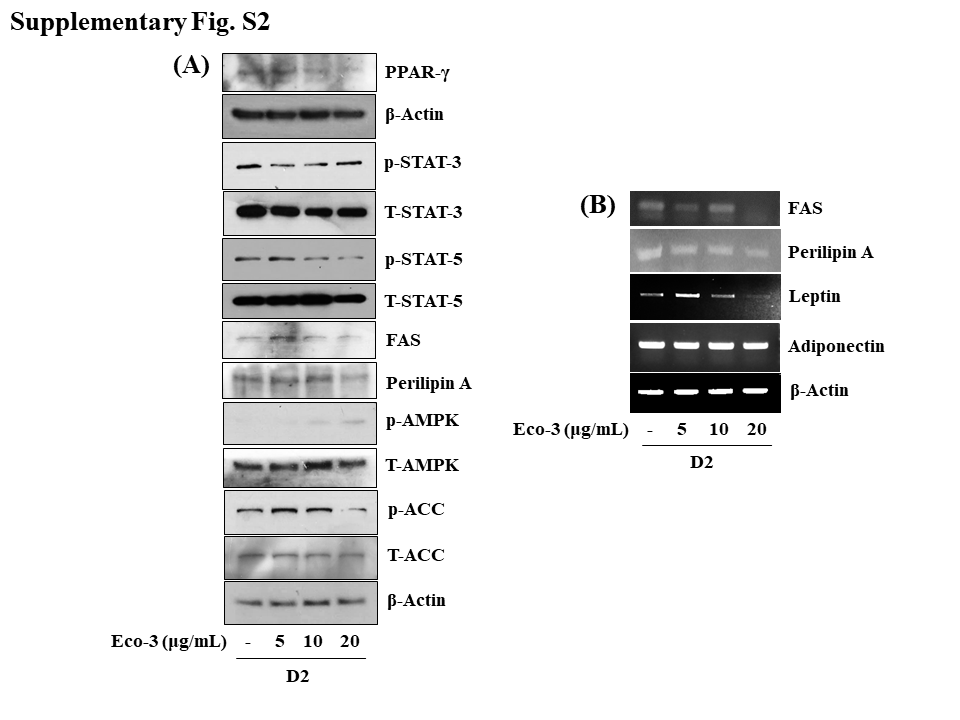

Supplement: Supplementary file 1 [file ijms-27-05394-s001.zip › Supplementary Figures/Fig. S2.TIF]

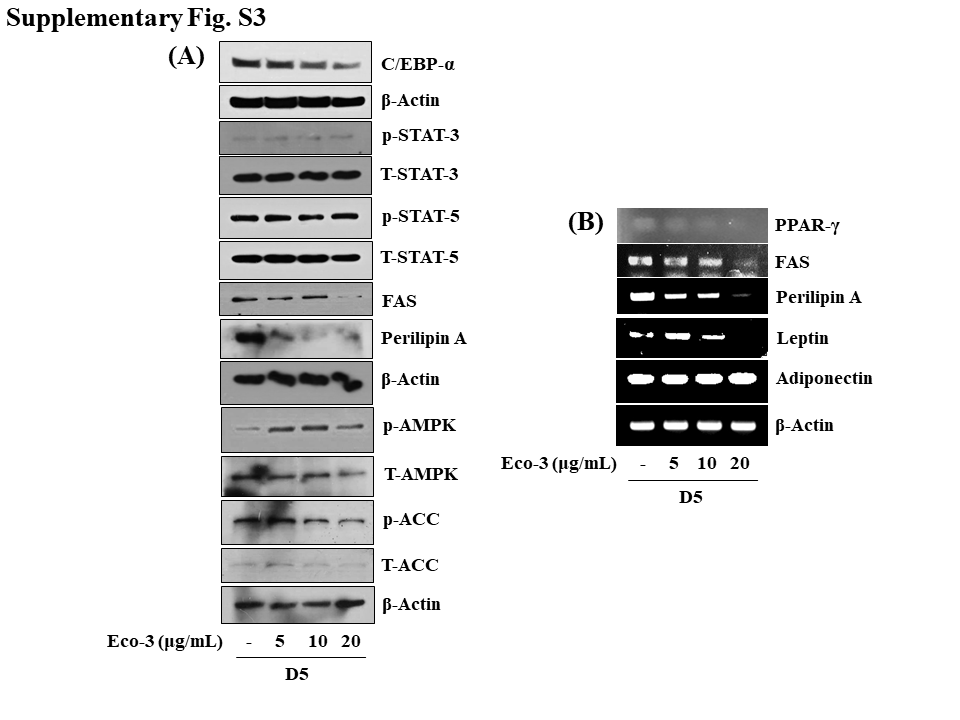

Supplement: Supplementary file 1 [file ijms-27-05394-s001.zip › Supplementary Figures/Fig. S3.TIF]
